# Dental Society Meetings

**Published:** 1882-08

**Authors:** W. C. Barrett

**Affiliations:** Buffalo, N. Y.


### ARTICLE II.

### *Dental Society Meetings,*

BY W. C. BARRETT, M. D., D. D. S., BUFFALO, N. Y.

That dentistry has made such rapid progress within the past two or three decades, is owing in great part to the beneficent influences of dental societies. Where, but a generation ago, our best men locked up within their own breasts the secret results of long experience or exhausting experiments, that they might enjoy their exclusive benefit, they now communicate their knowledge to their professional brethern freely, and as freely, and as freely receive in return. There are few, and they not usually among the best informed, who, rightfully belonging to a preceding age, yet hasten off to the Patent Office to secure the legal right to peddle out information, and make sure that the world shall not be the better for their having lived in it. There are some who even purloin the ideas which others would give to the public, and thus filch from the profession at large; but these last are usually mere hangers-on upon dentistry, who are not practitioners, and both classes are happily diminishing in numbers.

This satisfactory condition has been in great measure brought about by the dental societies which have been organized wherever a sufficient number of practitioners to form such a body could be periodically called together.

The continued association of dentists in the meetings of these bodies, has created an emulation as to who shall be able to lay before his fellows the greatest improvements in manipulation, the furthest advance in study and research, and the most direct and feasible method of reaching a desired end, or of overcoming an impending obstacle. The consequenee is that no field of practical science is more thoroughly cultivated than is ours. The histology and morphology of no part of the human anatomy is to-day better understood than that of the teeth and their integuments. But the rapid advance of a part of the profession has left far in the rear those of less studious habits or more sluggish understanding, so that in our societies to-day there are two classes of members—those who have outgrown the stale and standard topics of twenty years ago, and who demand more abstruse papers and discussions, and those who vote the time thrown away that is not devoted to what they call the "practical." Some way must be devised to satisfy the demand of both these parties, or dental societies will no longer serve the useful purpose which they have answered in the past. If the more advanced class, finding that they can derive but little benefit from the consideration of the stock subjects, gradually withdraw themselves, the profession as a whole will deteriorate; while if the younger and less experienced find nothing suited to their comprehension, they will lose all interest in the local societies, and progress will be arrested. Every dentist of broad views and a genuine love for his vocation, seeks not entirely selfish and personal benefits. He desires the good of the whole, and to this end he will labor. It only remains that he shall work intelligently. Those who have had years of experience should not lose sight of the struggles of their early days, but must aid the younger men to surmount the obstacles which were the greatest impediments to their own early advance. In the elementary societies, it is something worse than bad taste for the early practitioners to air their knowledge by pedantry, and the use of words of "learned length and thundering sound." Such pragmatical displays will not advance the cause which they profess to have at heart. Who that is in the habit of attending society meetings, has not heard the expressions of disgust and dissatisfaction at such waste of the opportunities of those who have, perhaps, at large sacrifice of time and convenience, come up to these meetings in the hope of obtaining useful knowledge? The wise essayist or speaker will endeavor to adapt his remarks to the average intelligence of his auditors, and he who fires far above their heads is contracting the usefulness of dental societies, and injuring the cause which he desires to serve. Let him reserve his abstruse theories and disquisitions until he finds himself among men who can appreciate and intelligently discuss such matters. There are few of us but can recall more than one instance when a meeting which promised usefulness was chilled and stricken to torpor by the interjection of some wild vagary of the imagination, or some absurdly recondite and subtile theory, nothing whatever akin to the professed aims of the society.

The older dentists can all remember many a problem, simple now to their experience, which sadly puzzled their unpractised understanding at the outset of their career. A rehersal in plain and direct language of the manner in which they overcame these difficulties, will not only be a pleasant retrospect to them, will not only fix their knowledge yet more firmly in their own minds, but will be of great interest and benefit to those who are now treading the very road over which they, long ago, painfully passed. Essays and addresses of this character, the material drawn, not from text books, but from the broader, richer, fresher fields of their own practical knowledge, may introduce subjects not commonly touched upon in mixed societies, and foster a love for study in those who are inclined to confine their researches and their practice to mere mechanical manipulation.

It was after some thought upon this subject, and to illustrate my point by personal application, that I accepted the position as essayist for the coming meeting of a local society, and formed the determination to step out of the beaten track, and to present something different from the convential society paper. The subject which I selected—Materia Medica—was one that in the estimation of the average country dentist is a dry one, but I thought that by treating it in an unusual manner it might perhaps be invested with interest. I commenced by reviewing a very few of the basal principles, spoke of general and local remehies then took up two or three of the principal ones in each of the four classes of Stimulants, Sedatives, Antispasmodics, and Anæsthetics, and very briefly described them. I then attempted a practical application by conducting a quiz, introducing an imaginative patient, describing a fictitious train of symptoms, and asking what should be the course of treatment. All the complications were those which I had good cause to remember as having arisen at different times in my own practice, and I found they were by no means strangers to the members present, nor were many of them entirely clear as to the best course of procedure under the circumstances. From their suggestions I I learned much myself, and I am sure that they in turn were benefited by the free interchange of opinion. At the risk of seeming rather to assume the pedagogue, I propose here to give a part of the questions propounded, with the answers which were finally agreed upon as proper, in the hope that they suggest to others a way for making such meetings beneficial. I commenced thus:

A young lady presents herself to you for the first time and is suddenly seized with a difficulty in breathing; not so severe as to demand instant remedial measures, but sufficient so to interfere with a proposed operation. What should be the first step?

Answer. Inquire if she be subject to such attacks.

If you find that she is what does it indicate?

A. Some probable organic difficulty; disease of the heart, or possibly aneurism.

How will it affect your proposed operation?

A. It will be necessary to proceed with extreme caution.

What will you do if the difficulty increases?

A. Place the patient in a recumbent position, in fresh air. See that the clothing be loose. Let her breath nitrite of amyrl, or ammonia. Use friction and give stimulants.

Would you give chloroform in such a case?

A. No. It would be especially dangerous.

If you find the patient is not subject to such attacks, to what might you propably attribute it ?

A. Nervousness; hysteria.

What remedies would you use ?

A. Sedatives and neurotics; camphor. The same directions as to position and clothing as in the former case.

How will you determine if a patient be fit to take an anæsthetic; chloroform for instance ?

A. Inquire concerning faintings and dyspnœa, or alcoholism. Listen over the precordial region and see if the rythm of the heart be regular and clear.

How if you wish to give ether ?

A. Examine the lungs, and see that the breathing be uninterrupted and regular.

If in the first steps of giving an anæsthetic, chloroform, for instance, your patient gets into a state of great excitement, would you push the administration ?

A. By no means; suspend operations entirely, or give more air until quiet be restored.

If after the patient becomes unconscious from chloroform the circulation is interfered with greatly, the lips become blue and collapse seems imminent, what would you do ?

A. Place her in a recumbent position in a current of fresh air, pull the tongue forward with a pair of forceps or with a hoe excavator that you may be sure the glottis is open, and then administer some kind of a shock to the nervous system—preferably an electric shock—but if a battery be not at hand use cold water, or a sudden concussion to start the heart into action, for its stoppage is the main source of danger. Make the patient breathe the fumes of nitrite of amyl.

How if the anæsthetic be sulph. ether ?

A. Place her in a recumbent position as before, see that the clothing be loose and the glottis open. Send an electric current along the course of the phrenic nerve. Get up artificial respiration. In either case see that the bodily heat is kept up.

What is the principal source of danger in chloroform narcosis?

A. Stoppage in the circulation.

In ether?

A. Stoppage of respiration. Remedial measures should be primarily directed to start into action the heart and lungs respectively.

How would you set about artificial respiration?

It was here ascertained that not one of those present had ever witnessed the process, or had an intelligent idea of the manipulations necessary. The action of the principal respiratory muscles was explained, and the movements necessary to stimulate their activity, when a volunteer patient presented himself, Marshall Hall's and Dr. Silvestre's methods were illustrated. The necessity for dispatch was explained, and to give the necessary elevation to the chest the operator's coat was off, rolled up and in place, the supposed patient was placed in position upon it, and was perforce breathing synchronously with the movements in less than five seconds. A great deal of interest was manifested in this demonstration, and all became thoroughly familiar with the necessary movements.

If in the administration there be great nervous depression, what remedies are indicated?

A. Stimulants; alcohol, brandy, whiskey.

How if the patient cannot be induced to swallow?

A. Rub alcohol into the circulation through the skin.

If there be no stimulant at hand what would you do?

A. Apply friction to the extremities.

If in the administration of any anæsthetic the lips become livid, what is the probable cause?

A. Asphyxia.

What is the remedy?

A. Remove the anæsthetic and assist respiration.

After the administration of an anæsthetic and the recovery of conscience, if there be considerable depression and a tendency to sleep, what would yon do?

A. Keep the patient in motion and assist the circulation by the administration of stimulants, remembering that the agent is in the blood, and must be eliminated mainly through the lungs, as quickly as possible.

Many incidental questions were asked by the members present, and details were demanded which it is unnecessary to repeat here, as I have endeavored only to set down the most concise answers returnable, and have made no attempt to sketch the sometimes extended debate over a query. The best method for the administration of chloroform and ether was discussed, and the necessity for something better than a folded napkin to apply to the face was pointed out. The dentists were reminded that while an atmosphere charged with four or five per cent. of chloroform might be breathed with impunity, ten or twelve per cent. especially in the earlier stages of narcosis, might result fatally. Almost any gas or vapor may be inhaled without special danger if it be sufficiently diluted. If a specific effect be desired this is best secured by giving the agent in specific quantities, and to this end, unless the operator has great experience and uses extreme caution, some good inhaler, which insures thorough dilution of the vapor, is essential to successful administration.

It can be readily seen that there are many emergencies in dental practice which might form the subject of a properly conducted quiz, and which would prove of especial interest to the younger practitioners. There will, in such a meeting, be a constant tendency to desultory discussion, which the conductor must have sufficient firmness to check. Long-winded "incidents," and personal reminiscences of cases interesting only to the relater, trivial circumstances, and the detailing of wonderful results as the consequence of the use of empirical remedies on the part of members, if allowed, will destroy the interest in any discussion. If the question under consideration be kept steadily in view, and every member be appealed to for concise and terse answers to the questions propounded, it is not difficult to make a meeting of this kind exceedingly profitable to every one.—*Independent Practitioner.*

